# A novel physics-based model for fast computation of blood flow in coronary arteries

**DOI:** 10.1186/s12938-023-01121-y

**Published:** 2023-06-12

**Authors:** Xiuhua Hu, Xingli Liu, Hongping Wang, Lei Xu, Peng Wu, Wenbing Zhang, Zhaozhuo Niu, Longjiang Zhang, Qi Gao

**Affiliations:** 1 Department of Radiology, Sir Run Run Shaw Hospital, School of Medicine, Zhejiang University, Hangzhou, China; 2 Hangzhou Shengshi Science and Technology Co., Ltd., Hangzhou, China; 3 The State Key Laboratory of Nonlinear Mechanics, Institute of Mechanics, Chinese Academy of Sciences, Beijing, China; 4 Department of Radiology, Beijing Anzhen Hospital, Capital Medical University, Beijing, China; 5 Biomanufacturing Research Centre, School of Mechanical and Electric Engineering, Soochow University, Suzhou, Jiangsu China; 6 Department of Cardiology, Sir Run Run Shaw Hospital, School of Medicine, Zhejiang University, Hangzhou, China; 7 Department of Cardiac Surgery, Qingdao Municipal Hospital, Qingdao, China; 8 Department of Medical Imaging, Jinling Hospital, Medical School of Nanjing University, Nanjing, Jiangsu China; 9 Institute of Fluid Engineering, School of Aeronautics and Astronautics, Zhejiang University, Hangzhou, China

**Keywords:** Coronary computed tomography angiography, Fractional flow reserve, Computational fluid dynamics, Physics-based fast model

## Abstract

Blood flow and pressure calculated using the currently available methods have shown the potential to predict the progression of pathology, guide treatment strategies and help with postoperative recovery. However, the conspicuous disadvantage of these methods might be the time-consuming nature due to the simulation of virtual interventional treatment. The purpose of this study is to propose a fast novel physics-based model, called FAST, for the prediction of blood flow and pressure. More specifically, blood flow in a vessel is discretized into a number of micro-flow elements along the centerline of the artery, so that when using the equation of viscous fluid motion, the complex blood flow in the artery is simplified into a one-dimensional (1D) steady-state flow. We demonstrate that this method can compute the fractional flow reserve (FFR) derived from coronary computed tomography angiography (CCTA). 345 patients with 402 lesions are used to evaluate the feasibility of the FAST simulation through a comparison with three-dimensional (3D) computational fluid dynamics (CFD) simulation. Invasive FFR is also introduced to validate the diagnostic performance of the FAST method as a reference standard. The performance of the FAST method is comparable with the 3D CFD method. Compared with invasive FFR, the accuracy, sensitivity and specificity of FAST is 88.6%, 83.2% and 91.3%, respectively. The AUC of FFR_FAST_ is 0.906. This demonstrates that the FAST algorithm and 3D CFD method show high consistency in predicting steady-state blood flow and pressure. Meanwhile, the FAST method also shows the potential in detecting lesion-specific ischemia.

## Introduction

Coronary artery disease (CAD) is the dominant reason for cardiovascular disease (CVD) death, with a mortality rate of 43.2% [1]. Invasive coronary angiography (ICA) [2] and non-invasive coronary computed tomographic angiography (CCTA) [3] are commonly used for evaluating coronary stenosis in traditional anatomy, whereas they have limitations when quantitatively assessing the myocardial ischemia caused by stenosis [4]. As a functional index for estimating coronary blood flow, fractional flow reserve (FFR) has been considered the “gold standard” for assessing coronary ischemia [5]. However, the application of FFR is still relatively low since it relies on invasive and costly catheterization that can possibly induce trauma by use of adenosine injection for hyperemia [6]. Due to the possibility of adoption of the computational fluid dynamics (CFD) method in diagnosing disease and guiding treatment options, patient-specific modeling of hemodynamics has been getting widely discussed for its importance [7–9]. The simulation methods have the potential to predict the progression or regression of lesions and provide guidance of treatment in pre- or post-operation when combined with anatomical and functional information [10]. Considering the cost of healthcare, it would be great if this process could be implemented for the cardiac catheterization laboratory procedures. At the same time, the diagnostic results can be achieved very efficiently with a low computational cost.

In recent years, the interest in the investigation of blood flow in the human arterial system using reduced-order models has been growing. One-dimensional (1-D) Navier–Stokes (NS) equations or variants of the equations were applied to solve the blood flow in synthetic or simplified arteries with various traditional schemes [11–13]. Since peripheral compliances and resistances significantly influence blood flow in arteries, patient-specific lumped parameter networks or zero-dimensional (0-D) models were coupled in the boundaries of 1-D models to simulate the microcirculation [14, 15]. However, a relationship between pressure and area is required to close the 1-D NS equations, which will introduce a set of coefficients accounting for physical and mechanical characteristics of the vessel. These parameters depend on the particular mechanical model chosen and vary as the targeted vessel changes. Additionally, these investigations place more emphasis on the normal blood flow and pressure in a reduced system rather than on clinical index and hemodynamics features caused by lesions in arteries. With the increase in the number of CAD patients in recent years, the fast and accurate prediction of blood flow in the coronary artery is getting imperative.

Recently, coronary artery blood flow modeling has emerged, starting from the analytical model to the machine-learning method. Huo et al. [16] proposed an analytical model derived from energy conservation to calculate the energy loss along stenosis in a tube-like model while measuring the blood flow as an input variable. Schrauwen et al. [17] made a pressure-drop prediction based on geometrical features about a few segments of coronary arteries using a plug flow velocity profile as an inlet boundary. In a subsequent study, Schrauwen et al. [18] proposed another method to quickly and accurately calculate pressure drop on the basis of a geometry and axial velocity profile assumed to be parabolic. They validated this method on straightened coronary arteries. However, for both studies, the velocity profiles on patient-specific models might be significantly different. Itu et al. [19] developed a patient-specific coronary flow model coupled with a lumped heart model and a physiological model for the microvascular bed. This reduced-order model enabled the on-site computation of blood flow; furthermore, the applicability of this model in FFR prediction has been validated in several investigations [20–24]. Instead of coupling one arterial segment directly to a terminal Windkessel, Boileau et al. [25] proposed an open-loop model where the systemic circulation was replaced with two 1D components coupled together through a lumped compartment and terminated with the usual peripheral three-element Windkessel model. The performance was validated using six cases with measured quantities from the published paper. In a new study carried out by Boileau et al. [26], intramyocardial pressure and material properties of the arterial wall were considered for a reduced-order model. The performance was assessed on a virtual cohort of 30 coronary artery stenoses. Although some order-reduced techniques simplify the sophisticated three-dimensional (3D) CFD simulation, they are still time-consuming. Meanwhile, the performance of a portion of models was not validated in patient-specific coronary arteries, or the number of validation cohorts was limited. More recently, Itu et al. [27] offered a machine-learning (ML) based approach to predict the index of myocardial ischemia. The model was trained using an extensive database of synthetically generated coronary anatomies, and the performance was assessed by comparing the predictions against the reduced-order model and against invasively measured FFR for 125 lesions. From then on, the machine-learning methods were gradually applied to predict the functional severity of lesions [28–30]. With these methods, the computation time is dramatically shortened. However, the data-driven model is usually questioned for the limited training datasets and lack of physical constraints. To overcome the aforementioned shortcomings and concerns, a physics-based fast model would be very valuable for quickly calculating blood flow, pressure, and other useful hemodynamic metrics in clinical practice.

In this study, a fast novel physics-based model is proposed to predict the blood flow and the pressure on-site, which is called FAST for convenience. This fast physics-based framework can be convenient for diagnosing coronary disease since the clinician can acquire the clinical index of concern before invasive procedures by relying on extensive clinical experience in medical imaging and little professional knowledge in fluid mechanics. The clinical quantity calculated is FFR measured from a guide wire-based procedure that can accurately measure blood pressure across coronary artery stenosis. FFR is a time-averaged pressure drop-related parameter and is defined as the ratio of pressure distal to a stenosis to the pressure proximal to the stenosis. Non-invasive CCTA derived FFR (CT-FFR) based on 3D CFD methods has shown advantages in determining myocardial ischemia, guiding therapeutic strategies, and providing prognostic assessment [31]. Thus, the 3D CFD-based approach FFR (FFR_CFD_) is computed to validate the feasibility of our FAST algorithm [32]. More importantly, invasive FFR was introduced to validate the diagnostic performance of the FAST method in detecting lesion-specific ischemia as a reference standard. The framework we proposed derives from the basic principle of the viscous fluid flow on a 1D round pipe and can quickly predict the blood flow and pressure with lower hardware requirements. The physics-based fast FFR (FFR_FAST_) is presented to assess the severity of myocardial ischemia induced by coronary stenosis. This method is expected to significantly reduce the calculation time and even to realize real-time calculation.

The paper is organized as follows. In “Results” section, we demonstrate the feasibility of FAST prediction against 3D CFD simulations over a quantity of patients’ data. We further validate the performance of FAST method with invasive FFR values among 402 lesions. In “Discussion” section, we discuss the differences of our proposed method from other studies with regard to a few considerable points. In “Conclusions” section, a conclusion of this work is provided. In “Methods” section, we present the method of the physics-based fast model. We describe the patient-specific microcirculation resistance model and build the ideal coronary model which is used to replace the diseased artery when a lesion appears in the bifurcation position, especially in the proximal portion of daughter vessels.

## Results

### Validation protocol

For each patient, a constant mean aortic pressure of 100 mmHg was applied to model the inlet pressure of a coronary artery at rest [33–35]. Thus, the hyperemic aortic pressure that was used to estimate FFR turned to 90 mmHg. The patient-specific left ventricular myocardial mass (LVMM) was used to estimate the blood flow of a coronary artery at rest. This blood flow was further adopted to determine the patient-specific hyperemic resistance at the outlet. The values of the myocardial mass and blood flow are listed in Table 1 statistically.

**Table 1:** LVMM quantification from CCTA

	LVMM (g)	TCBF (L/min)	LCBF (L/min)	RCBF (L/min)
Male (*n* = 250)	139 ± 30	0.2186 ± 0.0692	0.1530 ± 0.0484	0.0656 ± 0.0218
Female (*n* = 95)	121 ± 31	0.1970 ± 0.0709	0.1379 ± 0.0496	0.0591 ± 0.0213
Right dominance (*n* = 241)	132 ± 32	0.2013 ± 0.0727	0.1227 ± 0.0424	0.0876 ± 0.0303
Left or co-dominance (*n* = 104)	128 ± 29	0.2055 ± 0.0675	0.1599 ± 0.0525	0.0457 ± 0.0150

Reported values are mean ± standard deviation for both male and female patients

**Fig. 1:**
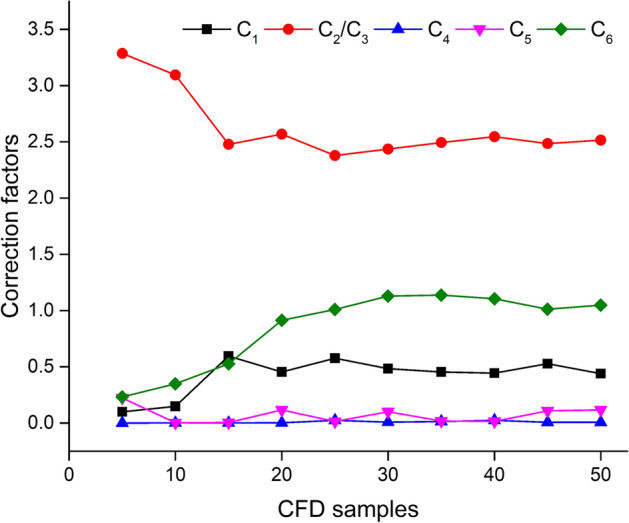
The convergence curves of six correction factors with the increase of CFD samples

3D CFD simulation was performed for all of the included patients. Then, multiple sets of CFD results were used to build the correction factors of pressure loss in the FAST method. The convergence curves can be seen in Fig. 1. Overall, it took 361 ± 174 iterations to enable the residuals of objective function to be less than 1e−6. It was found that the factors turned relatively stable after the number of CFD results reached 20. We tested the overall average pressure drop in 402 cases using seven sets of parameters, including CFD samples of 20, 25, 30, 35, 40, 45 and 50. The absolute errors among the result calculated using correction parameters with 20 CFD data and the results calculated from the other sets of parameters are all less than one percent. Furthermore, in order to use more data to assess the performance of the FAST method, the factors corrected by 20 CFD data were adopted and given in Table 2. Among 20 data, there are 13 results with FFR_CFD_ > 0.8 and 7 results with FFR_CFD_ ≤ 0.8.

**Table 2:** Correction factors in six pressure loss terms

Correction factor	C1	C2	C3	C4	C5	C6
Value	0.4544	2.5681	2.5681	0.0025	0.1169	0.9138

As a result, a total of 345 patients with 402 coronary lesions were available for comparative study. The mean age of the 345 patients was 61 (34–82) years and 243 (70%) were male. Among 402 vessels, 269 were left anterior descending (LAD) arteries, 9 were diagonal (D) arteries, 57 were left circumflex (LCx) arteries, 2 were obtuse marginal (OM) arteries, 62 were right coronary arteries (RCA) and 3 were posterior descending arteries (PDA). Among 402 coronary arteries, 137 vessels (34.1%) were identified as hemodynamically significant with invasive FFR of ≤ 0.8. Patient baseline characteristics are listed in Table 3. The performance of the FAST algorithm was evaluated by using both CFD results and invasive FFR.

**Table 3:** Baseline characteristics of the patients (*n* = 345)

Characteristic	Value
Age (yrs)	61 (34–82)
Gender M:F	250:95
Diabetes	93 (27.0%)
Hypertension	210 (60.9%)
Hyperlipidemia	126 (36.5%)
Current smoker	142 (41.2%)
Body mass index (kg/m^2^)	25.4 ± 3.0
Overall: 402 vessels	FFR ≤ 0.8: 137 vessels
LAD: 269	107
LCx: 57	14
RCA: 62	14
D: 9	2
OM: 2	0
PDA: 3	0

Values are mean ± standard deviation or *n* (%)

As is known, the drop of pressure in a coronary artery mainly arises from the size reduction of the luminal cross-section. Still, the contribution of the other terms is not distinct. In order to demonstrate the impact of each term, the contribution of each term to total pressure loss is counted. The stenotic region is always accompanied by the segments of constriction and expansion. Therefore, the pressure drops caused by constriction and expansion were added together to account for the effect of stenosis. The pressure loss at bifurcation regions was also counted. A total of 402 cases with 393,834 points were available and were used to evaluate the contribution.

FFR_CFD_ was evaluated at the downstream position far enough from the lesion to be considered irrelevant to the pressure recovery. Then, FFR_FAST_ was assessed at the same location. The diagnostic performance of FFR_FAST_ was estimated for all patients using accuracy, sensitivity, specificity, positive predictive value (PPV), negative predictive value (NPV), and with use of the area under the receiver’s operating characteristic curve (AUC) with a corresponding 95% confidence interval (CI) and a 0.8 threshold in both FFR_CFD_ and invasive FFR. Continuous variables were presented as mean ± standard deviation, and categorical variables were presented as totals or percentages. Because all samples were subject to non-normal distribution after the normality test, the Spearman correlation coefficient was used to assess the degree of correlation. Bland–Altman statistics were adopted to evaluate the difference. The error distribution was tested using the Shapiro–Wilk test. A pair-sample *t*-test was used to test the equivalence in bias. The standard deviation was tested using Chi-squared test. All statistical analyses were performed with the R programming language.

### Pressure loss of each term

**Fig. 2:**
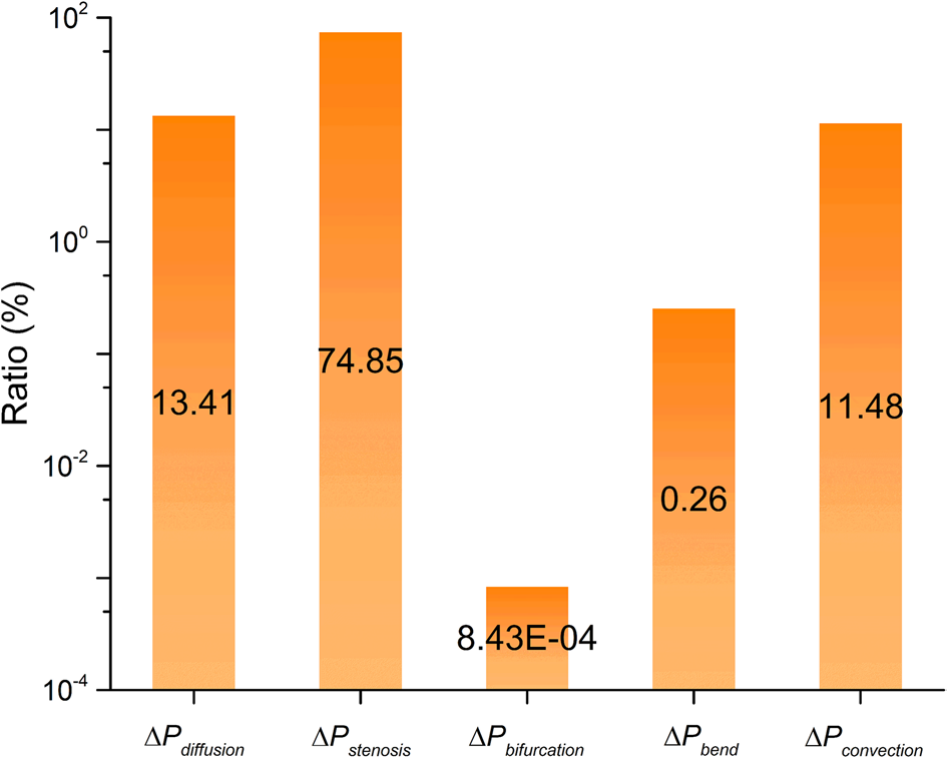
The ratio of pressure loss for five terms accounting for the overall pressure loss. The pressure drop resulting from stenosis reaches its highest level, with a value of 74.85%. In contrast, the effect of vessel bifurcation on overall pressure loss is negligible since the ratio is close to zero

A comparison of pressure drop among five loss terms is presented in Table 4. For 402 cases, the overall average pressure loss is 15.91 ± 65.59 mmHg. The average pressure loss for viscosity diffusion, cross-sectional area stenosis, vessel bifurcation, vessel bending and flow convection is 2.13 ± 3.59 mmHg, 11.91 ± 20.94 mmHg, 0.13e−4 ± 0.17e−3 mmHg, 0.04 ± 0.11 mmHg and 1.83 ± 62.38 mmHg, respectively. The ratio of pressure loss for five terms accounting for the overall pressure loss can be found in Fig. 2. The pressure drop resulting from stenosis reaches its highest, with a value of 74.85%, including constriction loss of 38.61% and expansion loss of 36.24%. The terms of viscosity diffusion and convection lead to a proportional pressure loss, corresponding to 13.41% and 11.48%. The pressure loss caused by morphological bending continues to decrease, with a small ratio of 0.26%. The effect of vessel bifurcation on overall pressure loss is negligible since the ratio is close to zero. The pressure loss accounts for 7.15% and 1.15% of bifurcation and bending in all local junction regions, while other pressure loss terms still make the most contributions to the pressure loss at those local regions.

**Table 4:** The total pressure loss and pressure loss of each term

Type	$\Delta P_{{{\text{total}}}}$	$\Delta P_{{{\text{diffusion}}}}$	$\Delta P_{{{\text{stenosis}}}}$	$\Delta P_{{{\text{bifurcation}}}}$	$\Delta P_{{{\text{bend}}}}$	$\Delta P_{{{\text{convection}}}}$
Mean (mmHg)	15.91	2.13	11.91	0.13e−4	0.04	1.83
Standard deviation (mmHg)	65.59	3.59	20.94	0.17e−3	0.11	62.38

Reported values are mean and standard deviation

### Performance by comparison with FFR_CFD_

**Fig. 3:**
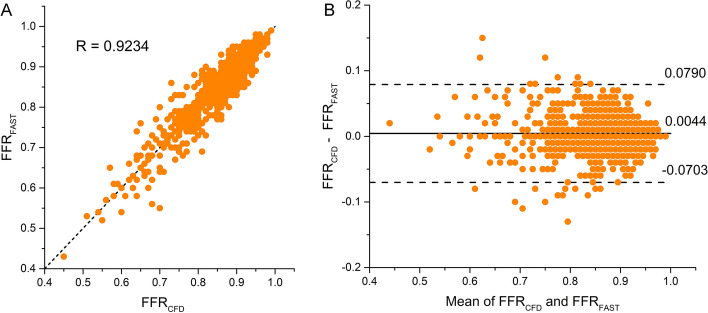
**A** Scatter plot of FFR_FAST_ and FFR_CFD_ with a correlation of 0.9234 (95% CI 0.9052 to 0.9373) (*P* < 0.000001). **B** Bland–Altman analysis plot of FFR_FAST_ and FFR_CFD_ with a mean difference of 0.0044 (95% limits of agreement: − 0.0703 to 0.0790)

Correlation between FFR_FAST_ and FFR_CFD_ is 0.9234 (95% CI 0.9052 to 0.9373) (*P* < 0.000001), and the slope and intercept are 0.9420 and 0.0439, respectively. The bias is 0.0044 with a standard deviation of 0.0381 (95% limits of agreement: − 0.0703 to 0.0790) in the Bland–Altman analysis, as shown in Fig. 3. The *p*-values are both < 0.001 when using the pair-sample *t*-test for bias and the Chi-squared test for standard deviation.

The performances of FFR_FAST_ on a per-vessel level assessment are listed in comparison with FFR_CFD_ in Table 5. The numbers of true positives, false positives, true negatives and false negatives are 119, 18, 250 and 15, and the diagnostic accuracy, sensitivity, specificity, PPV and NPV are 91.9% (95% CI 0.886–0.942), 88.8% (95% CI 0.819–0.934), 93.3% (95% CI 0.894–0.959), 86.9% (95% CI 0.798–0.918) and 94.3% (95% CI 0.906–0.967). The AUC is 0.967 (95% CI 0.951–0.983).

**Table 5:** Performance of FFR_FAST_ in patients on a per-vessel level in comparison with FFR_CFD_ (*n* = 402)

	FFR_FAST_ ≤ 0.80	95% CI
True positive, no.	119	–
False positive, no.	18	–
True negative, no.	250	–
False negative, no.	15	–
Accuracy (%)	91.9	(88.6, 94.2)
Sensitivity (%)	88.8	(81.9, 93.4)
Specificity (%)	93.3	(89.4, 95.9)
PPV (%)	86.9	(79.8, 91.8)
NPV (%)	94.3	(90.6, 96.7)

**Fig. 4:**
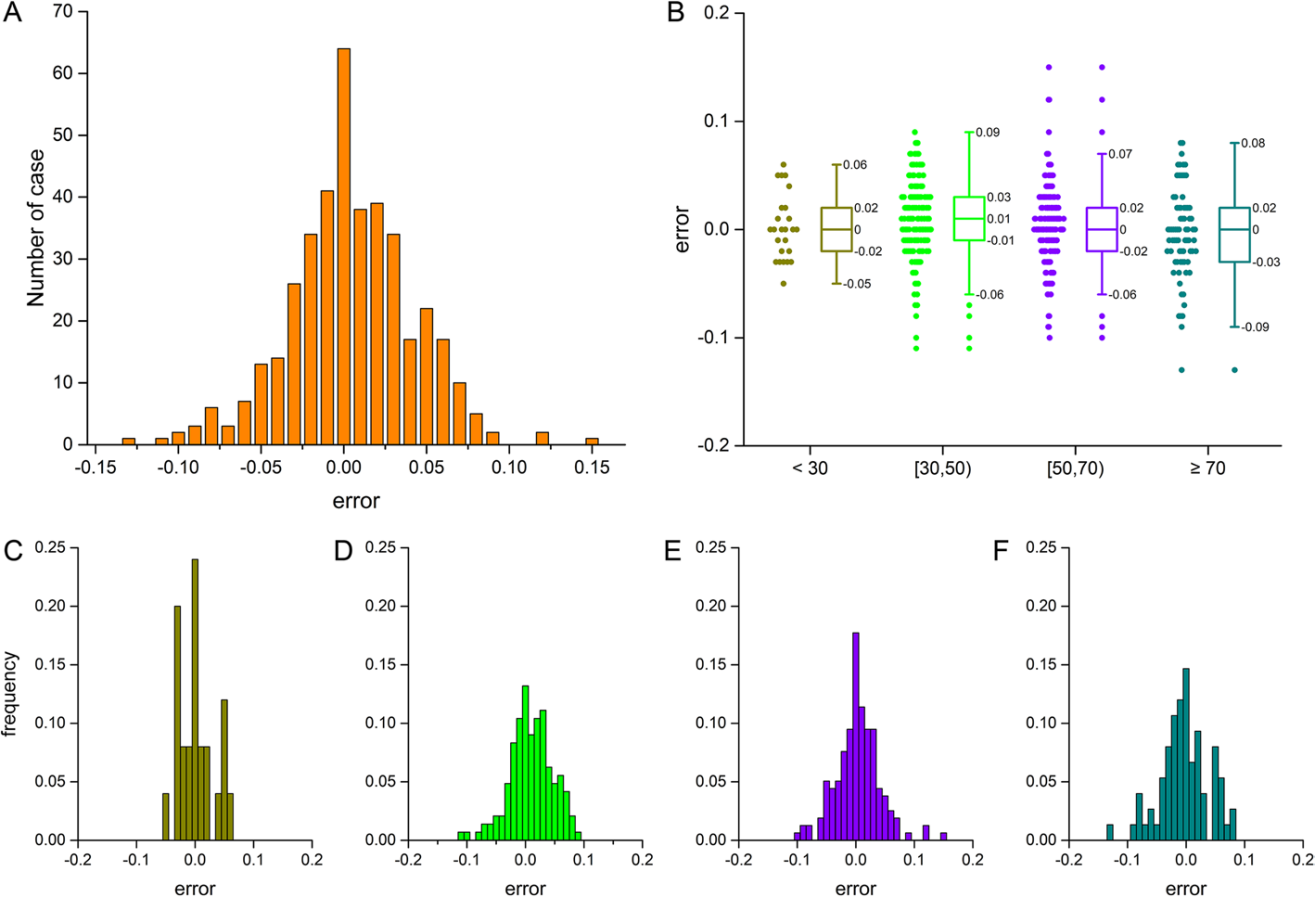
Illustration of error properties between FFR_FAST_ and FFR_CFD_ in different lesion severity groups. **A** The overall distribution of error. **B** Shows error distribution in different ranges. **C** to **F** Show the frequency of error in range of very mild < 30, mild [30, 50), moderate [50, 70) and obstructive ≥ 70, respectively

Based on stratification by lesion severity [36], the lesion among (0,100) is divided into four buckets: very mild < 30, mild [30, 50), moderate [50, 70) and obstructive ≥ 70. Table 6 summarizes the performance of the FAST algorithm in each bucket. For very mild lesions, the number of cases is 25. The bias in this bucket is 0.0020, and the standard deviation of error is 0.0300. For 144 mild lesions, a bias of 0.0099 and a standard deviation of error of 0.0370 are observed. For 158 moderate lesions, the bias is 0.0032 with a standard deviation of error of 0.0382. For 75 obstructive lesions, bias and standard deviation of error are − 0.0032 and 0.0414, respectively. Figure 4 demonstrates the error properties of the FFR_FAST_ in different lesion severity. The overall distribution of error is centered close to a positive mean value of < 0.01 (Fig. 4A). The error in the range of very mild lesions is more concentrative than the error in other lesions, and the maximum error of FFR in this range is 0.06 (Fig. 4C). The errors show the normal distribution in both very mild and obstructive lesions (*P* > 0.05).

**Table 6:** Performance of the proposed method categorized by lesion severity

Lesion severity	Sample size	Bias	Standard deviation	95% CI
Very mild	25	0.0020	0.0300	(− 0.0568, 0.0608)
Mild	144	0.0099	0.0370	(− 0.0625, 0.0824)
Moderate	158	0.0032	0.0382	(− 0.0716, 0.0781)
Obstructive	75	− 0.0032	0.0414	(− 0.0844, 0.0780)
Overall	402	0.0044	0.0381	(− 0.0703, 0.0790)

Bias, standard deviation and 95% confidence intervals are reported along with the sample size

**Fig. 5:**
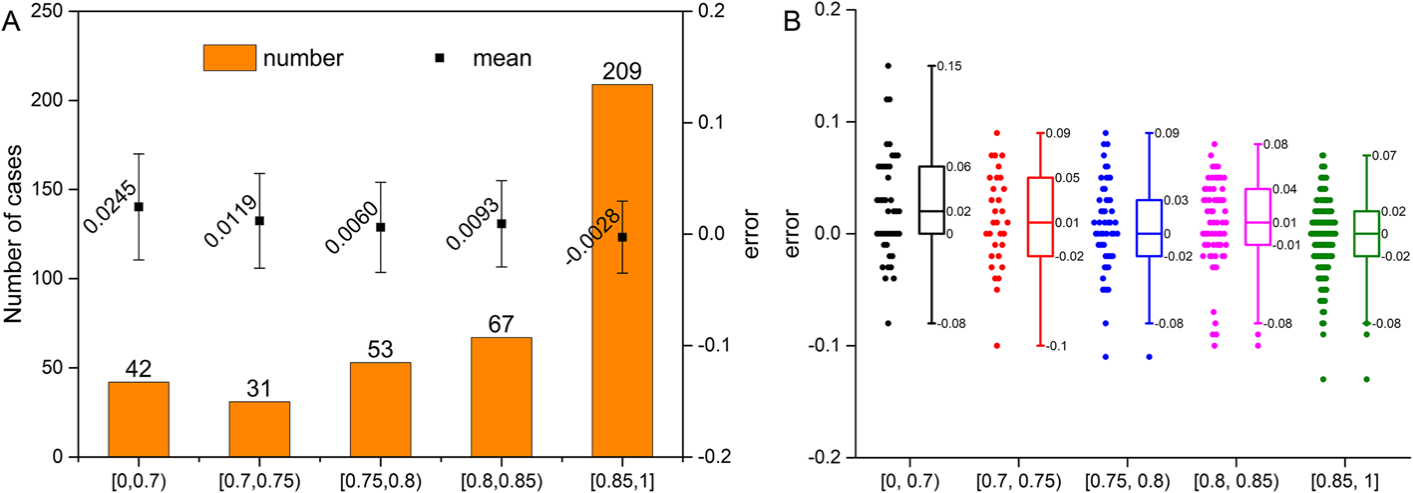
Illustration of error properties between FFR_FAST_ and FFR_CFD_ in different ranges of FFR values. **A** The number of FFR_FAST_ cases in different ranges and their error intervals. The errors of FFR are 0.0245 ± 0.0477, 0.0119 ± 0.0425, 0.0060 ± 0.0405, 0.0093 ± 0.0387 and − 0.0028 ± 0.0324 in different ranges of [0, 0.7), [0.7, 0.75), [0.75, 0.8), [0.8, 0.85), and [0.85, 1], respectively. **B** Error distribution in different ranges

Furthermore, according to the therapeutic strategies guided by the FFR value, the value from 0 to 1 was partitioned into five ranges. Figure 5A shows the performance of the FAST method in different ranges, in which the number of FFR_FAST_ occurrences was counted and the error bar was given. The mean error and the standard deviation are the smallest in the range of [0.85, 1], with a corresponding value of − 0.0028 and 0.0324 but with the largest number of cases of 209. The mean error and the standard deviation are the highest in the smallest [0, 0.7) range, with values of 0.0245 and 0.0477, respectively. The smallest number of cases is 31 in the range of [0.7, 0.75), with a bias of 0.0119 and a standard deviation of 0.0425. In the range of [0.75, 0.8), bias is 0.0060 and standard deviation of error is 0.0405, with a case number of 53. In the range of [0.8, 0.85), bias is 0.0093 and standard deviation of error is 0.0387, with a number of cases of 67. Figure 5B illustrates the discrete distribution properties of error in various scopes. The distribution of FFR_FAST_ in the range of [0, 0.7) appears with the maximum errors with the value of 0.15. It demonstrates that the FAST algorithm has a relatively large disparity in FFR prediction compared to the 3D CFD method. Error distribution shows a normal distribution in the ranges of [0, 0.7), [0.7, 0.75) and [0.75, 0.8). Outliers can be observed in the range of ≥ 0.75.

Generally, it takes around 3 min from imaging loading to FFR results for a single patient in the FAST algorithm. Specifically, it takes around 1.5 min to reconstruct the coronary artery model; for coronary artery discretization, it normally takes less than 1 min; for left ventricular myocardial mass calculation, it takes about 0.5 min; for the final step of calculating pressure distribution, which is the key step of the FAST algorithm, it statistically takes 2.79 ± 2.76 s based on all test cases. In contrast, only the computing time of the 3D CFD simulation is at least 1 h, considering computational time with four Inter (R) Xecon(R) CPU E3-1240 v5 processors for calculating blood flow and pressure.

### Validation against invasive data

**Fig. 6:**
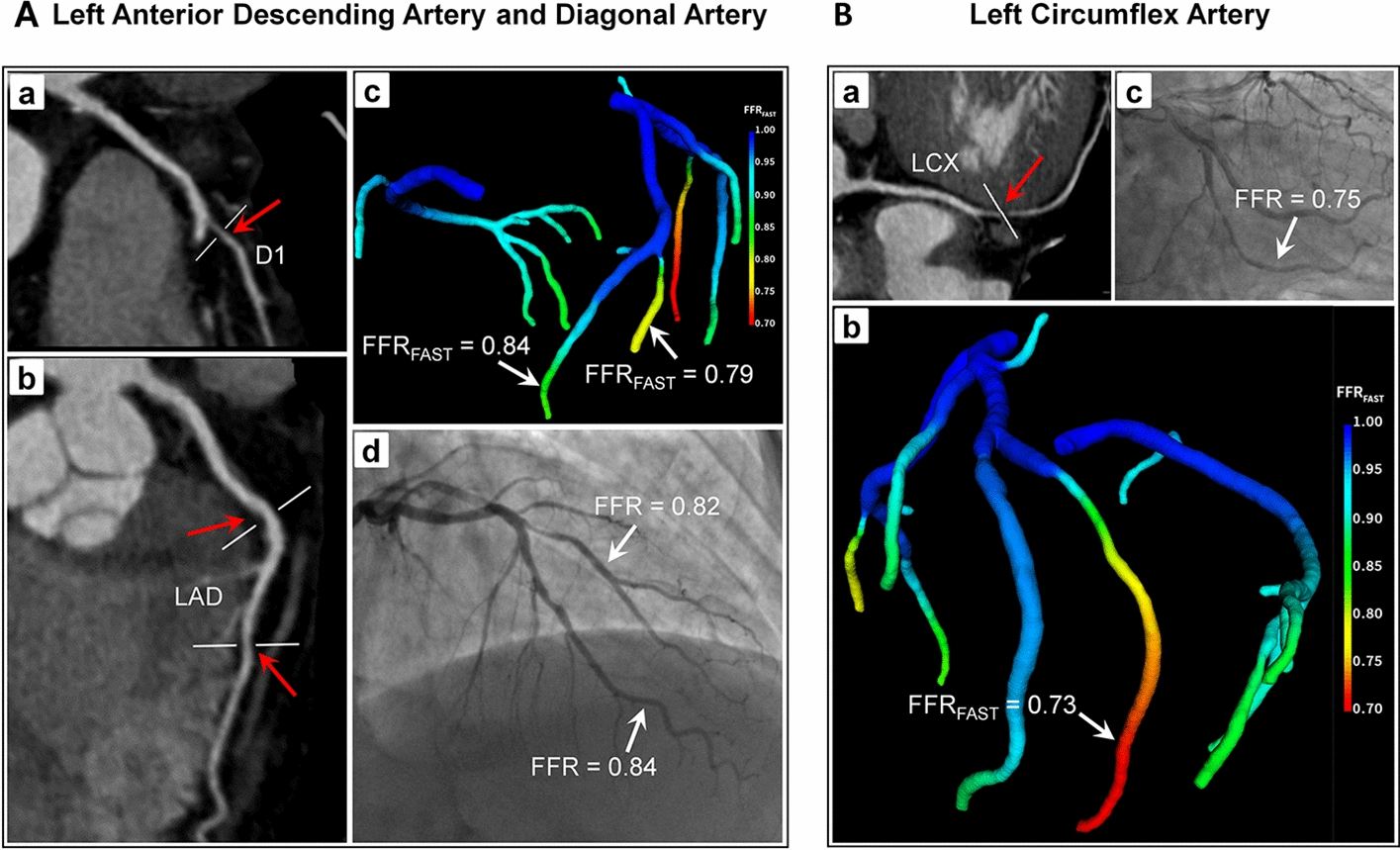
**A** Multiplanar reformat of CCTA demonstrates obstructive stenosis (red arrow) in the proximal portion of the D1 artery (**a**) and the middle portion of the LAD artery (**b**); computed FFR_FAST_ points to the distal of LAD and D1 (**c**); ICA and measured FFR in both LAD and D1 (**d**). **B** Multiplanar reformat of CCTA demonstrates obstructive stenosis (red arrow) in the middle portion of the LCx artery (**a**); computed FFR_FAST_ points to the distal of LCx (**b**); ICA and measured FFR (**c**)

In order to demonstrate the effectiveness in clinical practice, the FAST method was also validated using invasive FFR among 402 lesions, as shown in Table 7. The numbers of true positives, false positives, true negatives and false negatives are 114, 23, 242 and 23, respectively. The diagnostic accuracy, sensitivity, specificity, PPV and NPV are 88.6% (95% CI 0.849–0.914), 83.2% (95% CI 0.757–0.888), 91.3% (95% CI 0.871–0.943), 83.2% (95% CI 0.757–0.888) and 91.3% (95% CI 0.871–0.943). The AUC is 0.906 (95% CI 0.875–0.936). Similarly, the AUC of FFR_CFD_ is 0.920. As we can see from AUC, the performance of FFR_FAST_ is comparable to that of FFR_CFD_ (*P* < 0.000001). Figure 6 displays a representative example of FFR_FAST_ results compared to invasive FFR for a 60-year-old man with multivessel CAD. Two mild lesions were found in the LAD artery, and one moderate lesion was discovered in the D1 artery. In the LCx artery, a long serial lesion can be seen in the middle section. Three invasive FFR values were acquired behind the stenosis. If the value of invasive FFR is less than 0.8, it will be defined as significant ischemia. FFR_FAST_ shows non-significant ischemia, with a computed value of 0.83 in the LAD. ICA also demonstrates non-significant ischemia in LAD, with a measured FFR value of 0.84. FFR_FAST_ indicates significant ischemia with a computed value of 0.79 in the D1, but the ICA shows non-significant ischemia with a measured FFR value of 0.82. Both FFR_FAST_ and ICA demonstrate significant ischemia, with a computed value of 0.73 and a measured FFR value of 0.75, respectively. The corresponding values of invasive FFR, FFR_FAST_ and FFR_CFD_ can be found in Table 8.

**Table 7:** Performance of FFR_FAST_ in patients on a per-vessel level against invasive FFR (*n* = 402)

	FFR_FAST_ ≤ 0.80	FFR_CFD_ ≤ 0.80	*P* value
True positive, no.	114	114	–
False positive, no.	23	20	–
True negative, no.	242	245	–
False negative, no.	23	23	–
Accuracy (%)	88.6 [84.9, 91.4]	89.3 [85.8, 92.1]	0.60
Sensitivity (%)	83.2 [75.7, 88.8]	83.2 [75.7, 88.8]	1.00
Specificity (%)	91.3 [87.1, 94.3]	92.5 [88.4, 95.2]	0.49
PPV (%)	83.2 [75.7, 88.8]	85.1 [77.6, 90.4]	0.50
NPV (%)	91.3 [87.1, 94.3]	91.4 [87.2, 94.4]	0.94

**Table 8:** A representative comparison of values obtained with FFR_FAST,_ FFR_CFD_ and invasive FFR

FFR	FAST algorithm	3D CFD method	Clinical measurement
LAD	0.84	0.83	0.84
D1	0.79	0.81	0.82
LCX	0.73	0.74	0.75

## Discussion

The current study proposed a novel physics-based pressure drop approach combined with a microvascular resistance model for identifying the patient-specific hemodynamics information on site and in nearly real time. The total pressure loss along the coronary artery was estimated to be a linear combination of the pressure loss caused by viscosity diffusion, minor loss, and flow convection at each coronary artery part. Although a slight systematic bias of FFR_FAST_ can be seen from the Bland–Altman analysis, FFR_FAST_ shows decent consistency with FFR_CFD_. It demonstrates that the FAST algorithm shows greater accuracy compared to the 3D CFD method. More importantly, it illustrates that the performance of the FAST algorithm is nearly equivalent to that of the 3D CFD method in predicting hemodynamically significant information. The performance of the FAST method was further validated by dividing the lesion severity into four intervals. The smallest bias can be observed in the very mild interval. FFR_FAST_ appeared to underestimate FFR_CFD_ in very mild to moderate lesions, while it overestimated that in obstructive lesions to a large extent. Furthermore, performance of the FAST algorithm was also verified in different ranges of FFR values. The maximum bias was observed in a range of [0, 0.7). The previous work also showed the same phenomena [37]. This might result from the limited sample size in this particular region. Besides, the diagnostic performance of FFR_FAST_ in detecting lesion-specific ischemia was validated using invasive FFR as a reference standard. This work demonstrates the applicability of FFR_FAST_ for evaluating myocardial ischemia in a clinic. Meanwhile, this work straightforwardly illustrates that the FAST algorithm is comparable to that of the 3D CFD method in predicting hemodynamically significant information.

CCTA-based reduced-order algorithm and ML algorithm have also been applied to assess the functional significance of coronary ischemia in previous studies. With the use of reduced-order algorithm, the trials have shown an increase in accuracy from 74.6 to 83.9%, sensitivity from 77.8 to 87.5%, specificity from 59.3 to 86.8% and AUC from 0.83 to 0.88 in determining the hemodynamic significance of coronary stenosis [20, 22, 38]. For ML algorithm, diagnostic performance gradually has an improvement in accuracy from 82.0 to 91.8%, sensitivity from 61.0 to 97.6%, specificity from 76.7 to 94.0% and AUC from 0.86 to 0.96 [28–30, 39]. However, the sensitivity and specificity cannot show great results simultaneously in all researches. All of the aforementioned statistics were acquired on a per-vessel basis. Indeed, sensitivity and specificity discussion are always difficult in the presence of a referral bias where only patients undergoing the invasive measurement were included. The bias might increase false negative representation. However, this is a retrospective and analytical study. We have taken into consideration the referral bias in the design stage of our study. On a per-vessel level, our findings show a comparable result using FFR_FAST_ with accuracy of 88.6%, sensitivity of 83.2%, specificity of 91.3% and AUC of 0.906. This demonstrates that FFR_FAST_ is reliable and feasible as a promising tool for detecting lesion-specific ischemia.

The coronary artery model for 3D CFD computation can be obtained after the coronary artery was modeled and processed in the FAST algorithm, but this process is only a small part of the calculation for the 3D CFD method. More importantly, the luminal surface needs to be modified, and the mesh has to be generated before 3D CFD computation, which is time-consuming. Normally, the computation time for the 3D CFD method takes hours, which depends mainly on the number of processors. The approximate time taken to execute the FAST algorithm was much less with lower hardware requirements, which is almost a real-time process for FFR computation. It means that our FAST algorithm for blood flow prediction is potentially well-suited for a clinical setting since it can reduce the cost and improve the efficiency of clinical diagnoses at the same time. This technique has started a trial for clinical application in multiple centers in China.

The FAST algorithm for blood flow prediction was built based on a few key points. The first key point was that the physics-based fast model was derived from the differential equation of 1D viscous fluid motion. Six pressure loss terms, four terms related to morphological structure and two terms related to flow, were considered to depict the blood flow in a coronary artery. The coronary artery in this work was represented by the centerline which was composed of the successive micro-flow elements expressed by points and stored 3D coronary geometric information. As a result, the pressure drop was computed on each point through the superposition of pressure loss caused by each term. The total pressure drop was an integration of all the pressure loss of small short pipes along the centerline of the coronary arteries. Analogous with the method proposed in our method, an analytical model [16] was proposed for FFR computation derived from the conservation of energy, which considered various energy losses along the length of a factitious stenosis, namely the convective and diffusive energy losses as well as energy loss due to sudden constriction and expansion in the lumen area. This approach is similar to the present work; however, there are still some great differences. Firstly, the coronary artery tree is divided into many small vessel segments along the centerlines in FAST, and the pressure-loss model is applied to each vessel segment in the current work. The analytical model was validated by constructing constrictions in isolated arteries in vitro experiments and coronary arteries of eight swine in vivo experiments. Secondly, more factors causing pressure loss are considered in our approach, which is believed to be more sophisticated in estimating the pressure drop. Although the pressure drops caused by vessel bifurcation and bending are slight compared to total pressure loss, their contribution cannot be neglected for pressure loss in local regions. For example, the ratios of pressure loss are 7.15% and 1.15% for bifurcation and bending, respectively, when blood flows through the junction area mentioned in this paper. Thirdly, a boundary condition of microvascular resistance is applied to optimize pressure as a whole. It means that the present method can meet the patient-specific boundary conditions of the coronary artery. However, the analytical model needs to measure the flow velocity or give an empirical flow value. At last, the weighting coefficients of the FAST method are optimized from the dataset of high-fidelity 3D CFD results, which is more practicable for the computation of blood flow than the empirical coefficients used in the analytical model. In another work, the artery was modeled as a combination of tapered, stenosed, and curved models [17]. A formally uniform second-order polynomial of mean velocity with different fitting parameters was applied to characterize the pressure drops in each model. The total pressure drops were obtained by adding three independent pressure drops together. This method needed to provide mean velocity at the inlet of a coronary as a boundary condition as well. Then, an empirical formula acquired from previous studies was applied as the basic expression of pressure drop. The total pressure was then estimated through algebraic solving under a uniform boundary condition. In contrast, we expressed the pressure drop in a coronary artery based on the basic principle of the 1D viscous fluid flow. The pressure drop was predicted iteratively with patient-specific boundary conditions. Similarly, a zero-dimensional model was put forward [40] for the pressure drop across the stenosis, considering its geometric characteristics and flow rate. The results of this study showed similarities with 3D CFD simulation results, but the pressure drop caused only by stenosis and curvature was estimated by introducing the empirical relation and ML method. Our results demonstrate that the convection loss also dramatically impacts the pressure drop in the total coronary artery tree. Apart from occurring in the stenosis region, the convection loss could be triggered in the areas of bifurcation and bending. In addition, stenosis with irregular shapes might emerge in a coronary artery at any place [26]. For stenosis appearing at the bifurcation area, we indicate that the total pressure drop will be more accurate if the loss of bifurcation, loss of convection, and loss of bending caused by bifurcation could be taken into consideration.

Additionally, a patient-specific resistance boundary was applied to model the microcirculation of a coronary artery. A hyperemic aortic pressure was used as the inlet boundary condition of a coronary artery during estimation of FFR. According to Murray’s law in this paper, the total coronary blood flow was estimated while at rest based on LVMM and distributed to the branches. These two baseline coronary flow determination methods have been extensively used [41, 42]. Although different methods have different impacts on FFR prediction, none of the proposed methods in the study of Müller et al. resulted in a significant improvement of prediction error standard deviation [34]. In particular, the total volume of blood flow in left and right coronary arteries in this study was initially distributed based on the ratios of left and right coronary blood flow accounting for the total coronary blood flow. These two proportions were estimated from the measured coronary blood flow volume of a large sample of patients [43]. Despite the ratios being not patient-specific, they are critical for addressing the distribution of blood flow in left and right coronary arteries when the mathematical relationship between vessel size and flow rate, such as Murray's law, is apparently not suitable for this circumstance. Therefore, they were applied to split the total coronary blood flow into both left and right coronary arteries in this work. In particular, the proportions affect the flow distribution at each coronary outlet while at rest and further have an influence on microcirculation resistance. The previous study showed that the hyperemic factor was the most influential parameter for FFR prediction [44], but the study result obtained by Wilson et al. had been widely adopted to estimate the distal resistance reduction of each artery [45]. In the algorithm, therefore, coronary blood flow at each outlet was initially computed, and then patient-specific resistance at hyperemia was acquired at the corresponding outlet of the coronary artery. Furthermore, the flow was distributed at each bifurcation based on the root diameter of daughter vessels in this paper. Previous similar research utilized the average diameter computed over vessel nodes that were not marked as belonging to stenoses or bifurcation areas to split the flow at bifurcations [34, 46]. However, the diameters might be underestimated entirely if long stenosis had happened in an entire branch. In this case, hyperemic resistance at the outlet could be further misled since resistance was estimated from the flow of the outlet. Therefore, to model the appropriate boundary condition, an ideal vessel was also built to split the blood flow. The lesion occurrence could be anywhere in a coronary artery. The original stenotic vessel should be replaced by an ideal vessel to get a more reasonable distribution of flow if the stenosis appears in the proximal position of daughter vessels. For constructing the ideal vessel, two practical situations were considered. For a daughter vessel, the ideal diameter was first derived from its original diameter profile. The constructed ideal vessel might not be veritable because of the diversity of morphology and the difference caused by the ratio of lesion length to vessel length. On the other hand, the size of the daughter vessel is subject to the father vessel as well. Therefore, the ideal diameter was further optimized according to its father vessel. The ideal geometry can also be used to simulate the situation after a virtual percutaneous coronary intervention (PCI) procedure [37]. The stenosis can be located and identified based on the ideal diameter and original diameter. By replacing the diameter of the lesion position using the ideal one, the FAST method can provide an optional method to assess the blood flow condition before the interventional cardiologist performs the invasive procedure.

Regarding data-driven optimization of the algorithm, the ML-based model [27] is a net data-driven approach for predicting FFR as an alternative to the physics-based approach. This approach uses geometric features alone to estimate FFR without explicitly solving the hemodynamic equations. In order to train this model, a large synthetic database is generated using a reduced-order CFD model for quickly calculating the flow and pressure distribution for each coronary tree. In the FAST method, we also use 3D CFD results to optimize the weighting coefficients. This is a data-driven step taken to improve the accuracy and flexibility of our physics-based model. Different from the ML-based model, the FAST method needs a limited dataset for optimizing solutions. This database can be generated using a high-accuracy 3D CFD simulation. Thus, compared to the ML-based model, our FAST method introduces the physical information and constraints about coronary arteries to build the computational model and only needs a limited dataset to solve the unknown parameters simultaneously, which is more practicable and reproducible. Consequently, 20 CFD data were finally applied to solve the six free parameters in the FAST algorithm used in our approach. Despite a large number of CFD data that could be applied to regress those parameters, the correction parameters started to converge when the number of CFD samples reached 20. This phenomenon demonstrated that the physics-based model had a small dependency on the 3D CFD data. Admittedly, the parameters still have a small variation with the increase of the number of CFD samples, but the variation barely affects pressure estimation. These parameters were solved by the Trust-region-reflective (or other optimization algorithms) until they were converged to stable values. Then, the rest of the 345 CT data were used to validate this model. The great agreement among FFR_FAST_, FFR_CFD_ and invasive FFR shows that the FAST framework has good clinical applicability.

In the other aspects of the FAST method, the study showed that laminar flow was a reasonably good pattern for coronary blood flow. Meanwhile, CT-FFR estimated by the laminar flow model was closer to its invasive FFR [47]. Therefore, the laminar friction coefficient was chosen in this study. In addition, the steady-state simulation was fulfilled in the current study. The computation method cannot fully simulate the pulsatile coronary hemodynamics. However, invasive FFR gives a constant value computed as a ratio of the mean distal intracoronary pressure to the mean aortic pressure at the stage of the maximal hyperemia. Therefore, clinically practicable CT-based FFR normally follows the strategy of using steady approximation, which has been proved acceptable on its accuracy [46, 48]. This process can dramatically reduce the computation time. Consequently, the FAST method may provide a great convenience for FFR estimation in a real-time clinic. The previous study showed that the impact of prescribed inlet pressure, including pressure measured non-invasively, pressure acquired during invasive FFR measurement, and a fixed value of 100 mmHg, on FFR prediction frameworks is of little importance [34]. In the other two studies regarding the virtual functional assessment of coronary stenosis, a mean aortic pressure of 100 mmHg was also imposed at the inlet of coronary artery for CFD calculations [33, 35]. As a result, both virtual hemodynamic assessment models showed high diagnostic performance in detecting lesion-specific ischemia using invasive FFR as a reference standard. All studies illustrate that the statistical performance of FFR_FAST_ might not be changed in this paper even if patient-specific pressure was used. Therefore, a constant mean aortic pressure of 100 mmHg was used in this paper. Nevertheless, the patient-specific mean aortic pressure is recommended for the clinical setting to get more accurate results for a single patient in the future study.

One of the main limitations of this work is that the equivalent diameter in the centerline was applied to model the 3D coronary artery. The equivalent diameter cannot characterize the irregular configurations very well, especially the structure pattern in the stenotic region. It means that the pressure loss caused by these irregular shapes is neglected in FAST algorithm at present. In addition, a constant pressure loss coefficient of bifurcation is applied to the whole coronary tree. It will be more reasonable if the bifurcation-specific pressure loss coefficient can be used. Therefore, future work will focus on further optimizing the FAST algorithm to make it comparable with the 3D CFD method. Overall, according to the performance of the FAST algorithm compared with the traditional 3D CFD method and invasive FFR measurements, the usage of the fast novel physics-based model for the computation of FFR has achieved the purpose of taking the study further and to a larger extent.

## Conclusions

This work provides an alternative method to compute the blood flow of a coronary artery on site and almost in real time. The hemodynamic indexes we can obtain through this FAST framework include blood flow rate, pressure and FFR, which can offer a criterion for disease diagnosis and guide the treatment strategy. In addition to prognosis, the future work we can do is to simulate the hemodynamics after PCI or coronary artery bypass graft (CABG) using the FAST framework, which is helpful for a clinician when making therapeutic schedules and monitoring the postoperative recovery state. More importantly, the method we developed is suitable for predicting blood flow in the coronary artery and can be easily applied to other vascular systems with a large aspect ratio to model the patient-specific blood flow and predict the hemodynamic metrics.

## Methods

The morphological model of a coronary artery is reconstructed to simulate the blood flow using medical imaging data. The NS equations are used for 3D blood flow simulations to obtain the hemodynamic parameters, such as blood flow and pressure [9]. The equation of viscous fluid motion is applied to acquire the appropriate parameters for fast prediction of blood flow [49]. In this study, both 3D CFD blood flow simulation and the FAST prediction employ a pure microvascular resistance boundary condition for its calculations. More detailed information can be discovered in the following sub-sections.

### Study population

This is a retrospective and analytical study. Patients who had undergone both CCTA and invasive FFR measurements are included in this study. The retrospective analytical study protocol is approved by the Institutional Review Board (or Ethics Committee) of the Jinling hospital, and the requirement for written informed consent is waived. The identification of patients has been made anonymous. CCTA is performed using CT scanner platform (SOMATOM Definition Flash/Force and Definition AS+, Siemens Healthineers, Forchheim, Germany) with ≥ 64 detector rows. All CT images are reconstructed with a slice thickness of 0.6–0.75 mm and an increment of 0.5 mm. The major inclusion criteria were: (1) at least one stenosis in a major coronary artery with ≥ 2.0 mm diameter; (2) no occlusion in major coronary arteries; (3) no prior coronary stenting in major coronary arteries; (4) no prior CABG surgery in major coronary arteries; (5) no > 30% stenosis in left main coronary artery; (6) no serious image artifact in CCTA. A total of 365 patients are included in this study.

### Morphological model

CCTA images are processed using an in-house image processing algorithm. The main steps for the determination of the coronary artery are as follows:

- According to the first grey threshold, two seed points of the left and right coronary arteries are determined;
- Using a region growing algorithm, the ascending aorta and coronary artery tree comprised by a points cloud are segmented from CCTA images [50];
- The points cloud is corroded and inflated in sequence to detach the aorta from the integral model and to get the coronary artery model;
- Luminal surface of the coronary artery is generated and pre-processed as an input for CFD calculation. The centerline of the coronary artery is automatically extracted using the centerline extraction algorithm for the computation of the FAST algorithm [51]. In particular, the centerline is composed of discrete points which contain spatial coordinates and several coronary morphological information, such as diameter, cross-sectional area, curvature radius, etc. A bifurcation area is represented by one point connecting the parent and daughter points.

### Principle of pressure loss

Viscous fluid flow follows the differential equation of viscous fluid motion. If the flow is only subject to gravity and has an incompressible and steady-state flow, the differential equation can be integrated into Eq. 1 to depict the hydraulic loss in the flow direction of a 1D round pipe [49]:

1 $$\frac{{U_{1}^{2} }}{2g} + \frac{{P_{1} }}{\rho g} + Z_{1} = \frac{{U_{2}^{2} }}{2g} + \frac{{P_{2} }}{\rho g} + Z_{2} + \Delta h_{{\text{L}}} ,$$

where $i = 1,2$ represent two different cross-sections along a segment of the pipe, $U_{1}$ and $U_{2}$ are flow velocity, $P_{1}$ and $P_{2}$ are pressure, $Z_{1}$ and $Z_{2}$ are the position head, which is considered equal in a horizontal pipe, $\rho$ is the fluid density, $g$ is the acceleration of gravity, $\Delta h_{{\text{L}}}$ is hydraulic loss.

In addition to the Moody-type friction loss $h_{{\text{l}}}$ along the pipe, flow in a pipe also includes the local loss variable $h_{{\text{m}}}$ caused by the change of pipe shape, disturbance of flow velocity and direction, etc. Generally, $h_{{\text{l}}}$ is expressed by the Darcy–Weisbach equation for a pipe with a uniform flow:

2 $$h_{{\text{l}}} = \frac{\Delta P}{{\rho g}} = \lambda \frac{l}{d}\frac{{U^{2} }}{2g},$$

where $\Delta P$ is pressure loss, $U$ is average velocity, $\lambda$ is the friction coefficient along the pipe, $l$ is the length of the pipe and $d$ is the equivalent diameter of the pipe. For laminar flow, $\lambda { = }{{64} \mathord{\left/ {\vphantom {{64} {\text{Re}}}} \right. \kern-0pt} {\text{Re}}}$, and the Reynolds number ${\text{Re}} = {{\rho Ud} \mathord{\left/ {\vphantom {{\rho Ud} \mu }} \right. \kern-0pt} \mu }$, where $\mu$ is the blood dynamic viscosity coefficient. Local loss is commonly determined by the experiment, and it can be expressed as:

3 $$h_{{\text{m}}} = \frac{\Delta P}{{\rho g}} = \varsigma \frac{{U^{2} }}{2g},$$

where $\varsigma$ is the local loss coefficient. Based on the superposition principle of hydraulic loss, each loss term is considered separate without interference. Therefore, the total pressure loss as fluid flows through a pipe can be written as:

4 $$\Delta P = \rho g\left( {h_{{\text{l}}} + h_{{\text{m}}} + \frac{{U_{2}^{2} - U_{1}^{2} }}{2g}} \right) = \rho \lambda \frac{l}{d}\frac{{U^{2} }}{2} + \rho \varsigma \frac{{U^{2} }}{2} + \rho \frac{{U_{2}^{2} - U_{1}^{2} }}{2},$$

where the right-side terms represent the pressure loss caused by viscosity diffusion, minor loss, and flow convection, respectively.

### Physics-based fast model

**Fig. 7:**
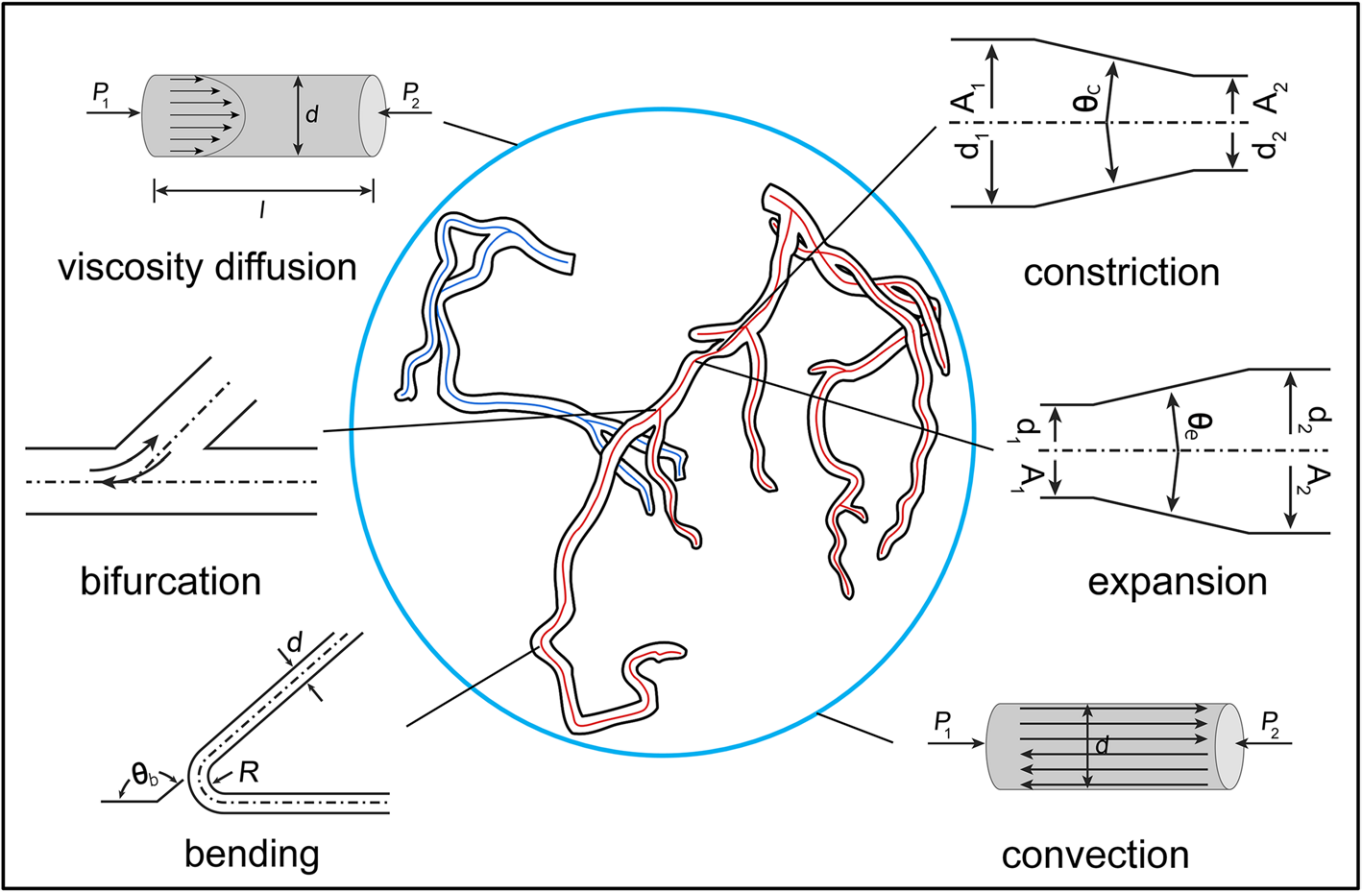
Illustration of six different types of flow patterns which are extracted from a 3D coronary model and applied in a coronary centerline for the computation of blood flow. Four flow patterns are in connection with geometric structure, and the other two occur as blood flows. $d$ represents the diameter of vessel, $A$ represents the cross-sectional area, and $P$ is the pressure. In particular, $\theta_{{\text{b}}}$ is defined as the amount of change in the angle of directional vector of centerline. $\theta_{{\text{c}}}$ and $\theta_{{\text{e}}}$ are, respectively, representing the changing degrees of the vessel diameter with constriction and expansion, which is determined by the diameters of two adjacent points and the distance between them

For the fast blood flow model, the key is to accurately estimate the pressure drop along the centerline of coronary arteries. In the present study, a long artery is supposed to comprise a number of successive discrete points. Two adjacent points are regarded as the start and end of one vessel part, which resembles a short pipe. In other words, the entire coronary artery tree consists of many consecutive short pipes. The length of the pipe is around 0.3 mm, which is the distance between two pixel points. Therefore, the total pressure loss, $\Delta P$, is an integration of all the pressure loss, $\Delta P_{i}$, of the small short pipes along the centerline of the coronary arteries, and the subscript $i$ indicates the index of small short pipes. The pressure at the* j*th point of the centerline $P_{j}$ is expressed as:

5 $$P_{j} = P_{0} - \sum\limits_{i = 1}^{j} {\Delta P_{i} } ,$$

where $P_{0}$ is the pressure at the inlet of the coronary artery. In fluid mechanics, the pressure loss along the pipe can be estimated as a linear combination of the pressure loss caused by viscosity diffusion ($\Delta P_{{i,{\text{diffusion}}}}$), cross-sectional area constriction ($\Delta P_{{i,{\text{constriction}}}}$), cross-sectional area expansion ($\Delta P_{{i,{\text{expansion}}}}$), vessel bending ($\Delta P_{{i,{\text{bend}}}}$) and flow convection ($\Delta P_{{i,{\text{convection}}}}$). For bifurcation areas, except for accounting for the pressure loss similar to that along the pipe, loss caused by vessel bifurcation ($\Delta P_{{i,{\text{bifurcation}}}}$) is introduced to sub-branches represented by separate pipes. A schematic description of six different pressure losses is shown in Fig. 7.

Therefore, the $\Delta P_{i}$ of a short pipe cell can be written as:

6 $$\Delta P_{i} = \Delta P_{{i,{\text{diffusion}}}} + \Delta P_{{i,{\text{constriction}}}} + \Delta P_{{i,{\text{expansion}}}} + \Delta P_{{i,{\text{bifurcation}}}} + \Delta P_{{i,{\text{bend}}}} + \Delta P_{{i,{\text{convection}}}} .$$

The empirical formulas of the right-hand terms in Eq. (6) are given in Eqs. (7) to (12):

7 $$\Delta P_{{i,{\text{diffusion}}}} = C_{1} \cdot \rho \lambda_{i} \frac{{l_{i} }}{{d_{i} }}\frac{{U_{i}^{2} }}{2},$$

8 $$\Delta P_{{i,{\text{constriction}}}} = C_{2} \cdot \rho \varsigma_{2i} \frac{{U_{i}^{2} }}{2},$$

9 $$\Delta P_{{i,{\text{expansion}}}} = C_{3} \cdot \rho \varsigma_{3i} \frac{{U_{i}^{2} }}{2},$$

10 $$\Delta P_{{i,{\text{bifurcation}}}} = C_{4} \cdot \rho \varsigma_{4i} \frac{{U_{i}^{2} }}{2},$$

11 $$\Delta P_{{i,{\text{bend}}}} = C_{5} \cdot \rho \varsigma_{5i} \frac{{U_{i}^{2} }}{2},$$

12 $$\Delta P_{{i,{\text{convection}}}} = C_{6} \cdot \rho \left( {\frac{{U_{{i,{\text{outlet}}}}^{2} }}{2} - \frac{{U_{{i,{\text{inlet}}}}^{2} }}{2}} \right).$$

The parameters $l_{i}$, $d_{i}$ and $U_{i}$, respectively represent the length, average diameter, and mean velocity of the *i*th local vessel segment, $U_{{i,{\text{outlet}}}}$ and $U_{{i,{\text{inlet}}}}$ are the outlet and inlet mean velocities at the *i*th vessel segment, $\rho$ is the density of blood, the parameters $\lambda_{i}$, $\varsigma_{2i}$, $\varsigma_{3i}$, $\varsigma_{4i}$ and $\varsigma_{5i}$ are the standard pressure loss coefficients, and $\left\{ {C_{1} ,C_{2} ,C_{3} ,C_{4} ,C_{5} ,C_{6} } \right\}$ are the correction factors of pressure drop. The pressure loss coefficient $\lambda_{i}$ is relative to the local Reynolds number. $\varsigma_{2i}$, $\varsigma_{3i}$, $\varsigma_{4i}$ and $\varsigma_{5i}$ relate to the geometric parameters (Table 9) [52]. Although the formulas are similar among different works, the coefficients in the equations are slightly different [49, 52, 53]. In this work, we modified the coefficients of some equations based on our tests.

**Table 9:** The detailed formulas for different kinds of pressure loss

Type	Formula	Coefficient of pressure loss
$\Delta P_{{i,{\text{diffusion}}}}$	$\rho \lambda_{i} \frac{{l_{i} }}{{\overline{d}_{i} }}\frac{{U_{i}^{2} }}{2}$	$\lambda_{i} = {{64} \mathord{\left/ {\vphantom {{64} {\text{Re}}}} \right. \kern-0pt} {\text{Re}}}_{i}$
$\Delta P_{{i,{\text{constriction}}}}$	$\rho \varsigma_{2i} \frac{{U_{i}^{2} }}{2}$	$$\varsigma_{2i} = \left\{ \begin{gathered} \frac{{\lambda_{i} }}{{8\sin ({{\theta_{{\text{c}}} } \mathord{\left/ {\vphantom {{\theta_{{\text{c}}} } {2)}}} \right. \kern-0pt} {2)}}}}\left[ {1 - \left( {\frac{{A_{2} }}{{A_{1} }}} \right)^{2} } \right],\;\theta_{{\text{c}}} < 30^\circ \hfill \\ \frac{{\lambda_{i} }}{{8\sin ({{\theta_{{\text{c}}} } \mathord{\left/ {\vphantom {{\theta_{{\text{c}}} } {2)}}} \right. \kern-0pt} {2)}}}}\left[ {1 - \left( {\frac{{A_{2} }}{{A_{1} }}} \right)^{2} } \right] + \frac{{\theta_{{\text{c}}} }}{1000},\;30^\circ \le \theta_{{\text{c}}} < 90^\circ \hfill \\ \end{gathered} \right.$$
$\Delta P_{{i,{\text{expansion}}}}$	$\rho \varsigma_{3i} \frac{{U_{i}^{2} }}{2}$	$\varsigma_{3i} = \frac{{\lambda_{i} }}{{8\sin ({{\theta_{{\text{e}}} } \mathord{\left/ {\vphantom {{\theta_{{\text{e}}} } {2)}}} \right. \kern-0pt} {2)}}}}\left[ {1 - \left( {\frac{{A_{1} }}{{A_{2} }}} \right)^{2} } \right] + \sin (\theta_{E} )\left[ {1 - \left( {\frac{{A_{1} }}{{A_{2} }}} \right)} \right]^{2}$
$\Delta P_{{i,{\text{bifurcation}}}}$	$\rho \varsigma_{4i} \frac{{U_{i}^{2} }}{2}$	$\varsigma_{4i} = 0.1$
$\Delta P_{{i,{\text{bend}}}}$	$\rho \varsigma_{5i} \frac{{U_{i}^{2} }}{2}$	$\varsigma_{5i} = \left[ {0.131 + 0.163\left( \frac{d}{R} \right)^{3.5} } \right]\frac{{\theta_{{\text{b}}} }}{90^\circ }$
$\Delta P_{{i,{\text{convection}}}}$	$\rho \left( {\frac{{U_{{i,{\text{outlet}}}}^{2} }}{2} - \frac{{U_{{i,{\text{inlet}}}}^{2} }}{2}} \right)$	–

### Patient-specific microcirculation

The circulation of a coronary artery consists of an epicardial coronary artery, pre-arteriolar vessel and arteriolar vessel. Normally, the coronary artery that can be observed through CCTA includes epicardial coronary arteries and a fraction of pre-arteriolar vessels. The majority of microcirculation vessels are covered by the myocardium. The reconstructed vessel seen in vitro by imaging data includes only the epicardial coronary arteries as well. The boundary condition is essential for numerical simulation; therefore, a lumped parameter network (Windkessel boundary condition) is generally used for modeling the flow–pressure relationship in the microvasculature of the coronary system [9, 54].

Because FFR is computed as a ratio of the mean distal intracoronary pressure to the mean aortic pressure at steady-state maximal hyperemia [2, 55, 56], pressure drop of blood flow through a coronary artery can be estimated at a steady state for both 3D CFD simulation and FAST prediction [57]. Therefore, the pressure at the inlet of the coronary artery could be set to a mean aortic pressure measured by the coronary catheter or estimated from the brachial arterial pressure of the patient. In general, pressure at the ostium in a coronary artery is considered to be equal to the aortic pressure. However, the pressure at the inlet of a coronary artery is reduced during hyperemia [45, 58, 59]. In our work, therefore, we account for this effect during FFR estimation in the algorithm, namely

13 $$P_{{{\text{a}},{\text{h}}}} = \omega P_{{{\text{a}},{\text{r}}}} ,$$

where $P_{{{\text{a}},{\text{h}}}}$ is the aortic pressure at hyperemia, $P_{{{\text{a}},{\text{r}}}}$ is the aortic pressure at rest, and $\omega$ is a hyperemic reduction parameter and set to be 0.9.

A patient-specific microvascular resistance model is adopted to characterize the pressure of the outlets at hyperemia. The pressure at the outlets $P_{k,h}$ is modeled as:

14 $$P_{{k,{\text{h}}}} = Q_{{k,{\text{h}}}} R_{{k,{\text{h}}}} + P_{{\text{v}}} ,$$

where $Q_{{k,{\text{h}}}}$ and $R_{{k,{\text{h}}}}$ are the flow and microvascular resistance related to the *k*th coronary branch at a hyperemic state and $P_{{\text{v}}}$ is the distal pressure of coronary microcirculation (central venous pressure). The critical step of the outlet boundary condition is how to get patient-specific $R_{{k,{\text{h}}}}$ at each outlet of the coronary artery.

According to allometric scaling laws, total coronary blood flow is proportional to left ventricular myocardial mass under resting conditions [60]. Therefore, total coronary blood flow was first estimated based on left ventricular myocardial mass. Then the proportions of left and right coronary blood flow accounting for the total coronary blood flow under different coronary artery dominances were used to straightforwardly distribute the total blood flow to the left and right coronary arteries [43]. The mathematical relationship between flow rate and vessel diameter can be expressed by $Q \propto d^{3}$ under certain conditions, such as constant wall shear stress and homeostatic level, based on Murray’s law [61], where $Q$ is the flow rate through a blood vessel at a resting state and $d$ is its diameter. This flow–diameter relationship can also be comprehended by Poiseuille’s law. Blood flows from the coronary entrance to the downstream arteries, and the distribution of blood flow for each vessel follows the rule (see Eq. 15) when it flows through bifurcation:

15 $$Q_{pn} = Q_{p} \frac{{d_{pn}^{3} }}{{\sum\limits_{n = 1}^{s} {d_{pn}^{3} } }}\left( {s \ge 2} \right),$$

where $p$ represents the attribute of the parent vessel, $s$ represents the number of sub-branches, and $n$ is an integral number. For daughter vessels, $d$ is the healthy diameter extracted from the first point in the centerline of the branch. Blood flow in all coronary branches can be distributed in this way while at rest.

Under resting conditions, blood flow is forced through each branch into the myocardium and reaches the right atrium by veins following the relationship:

16 $$\Delta P^{\prime}_{{k,{\text{r}}}} = Q_{{k,{\text{r}}}} \cdot R_{{k,{\text{r}}}} ,$$

where $\Delta P^{\prime}_{{k,{\text{r}}}}$ is the pressure difference between the distal of the epicardial conductive coronary artery and central vein, and $R_{{k,{\text{r}}}}$ is myocardial microcirculation resistance at rest. The pressure difference is formulated as:

17 $$\Delta P^{\prime}_{{k,{\text{r}}}} = P_{{a,{\text{r}}}} - P_{{\text{v}}} ,$$

where $P_{{a,{\text{r}}}}$ is the mean aortic pressure at rest and $P_{{\text{v}}}$ is set to 6.5 mmHg [62].

When the pressure difference and the blood flow in all coronary branches are identified, the resistance at rest can be determined based on Eq. 14. Wilson et al. [45] showed that coronary resistance at maximum hyperemia is reduced by 0.24 of the resting value. Therefore, patient-specific $R_{{k,{\text{h}}}}$ at hyperemia can be formulated by

18 $$R_{{k,{\text{h}}}} = 0.24R_{{k,{\text{r}}}} .$$

### Ideal model for a diseased artery

A lumped parameter network as a sophisticated model is applied to simulate the blood vascular system. The procedures used to get the reasonable value of parameters using this model, like the boundary resistance in our paper, is crucial for the prediction of hemodynamic parameters. In general, Murray’s law is used to depict the relationship between vessel size and blood flow. However, the flow allocation could lead to error if the reduction of the diameter occurs in the initial position of the daughter vessels [63]. One of the highlights of our work is building an ideal geometry (healthy profile) of a coronary artery to replace the diseased segment that appeared in the proximal region of the daughter vessels to model the rational resistance boundary condition for both 3D CFD simulation and the FAST prediction.

There are many paths for a coronary tree to have, from the ostium to the ends. One individual route comprised a few segments partitioned by the bifurcation. Different vessel segments in different paths are labeled in the algorithm. Taking a coronary tree as an example, the ideal shape of each independent path is built. The ideal diameter profile $d_{{{\text{ideal}},j}}$ is initially obtained by linearly fitting the original diameter profile $d_{{{\text{orig}},j}}$ using the least square method. Subsequently, the final ideal model is optimized globally through a nonlinear programming approach. In general, the diameter size in the current branch is subject to its parent branch, which is neither larger than the size of its parent branch nor smaller than that of its sub-branches. For each coronary artery segment, additionally, the diameter of the ideal model is regarded as monotonically non-increasing from the beginning to the end position [37]. Therefore, these two constraints (Eqs. 21 and 22) are introduced to correct the current branch diameter.

Overall, the ideal diameter should be closest to and not less than the original diameter profile. Therefore, the ideal diameter in one path of the coronary artery tree is automatically constructed using a minimum objective function and formulated mathematically as:

19 $$\begin{array}{*{20}c} {\min } & {f\left( {\alpha_{j} } \right)} \\ \end{array} = \sum\nolimits_{j} {\sqrt {\alpha_{j} \cdot d_{{{\text{ideal}},j}} - d_{{{\text{orig}},j}} } } ,$$

such that

20 $$\alpha_{j} > 0,$$

21 $$\alpha_{j} d_{{{\text{ideal}},j}} > \alpha_{j + 1} d_{{{\text{ideal}},j + 1}} ,$$

22 $$\chi_{j - 1,j} = \chi_{j,j + 1} ,\quad {\text{if}}\;j - 1\;{\text{to}}\;j + 1\;{\text{belong}}\;{\text{to}}\;{\text{an}}\;{\text{individual}}\;{\text{segment,}}$$

where $\alpha_{j}$ is a coefficient, $\chi_{j - 1,j}$ and $\chi_{j,j + 1}$ are slopes of the diameter profile for each vessel segment. Consequently, the ideal diameter for one path can be found when $\alpha_{j}$ is optimized. The ideal diameter in each track can be obtained by traversing all paths using this optimization algorithm. For a vessel segment that appeared in multiple courses, the largest diameters among all are applied to determine the final ideal size.

**Fig. 8:**
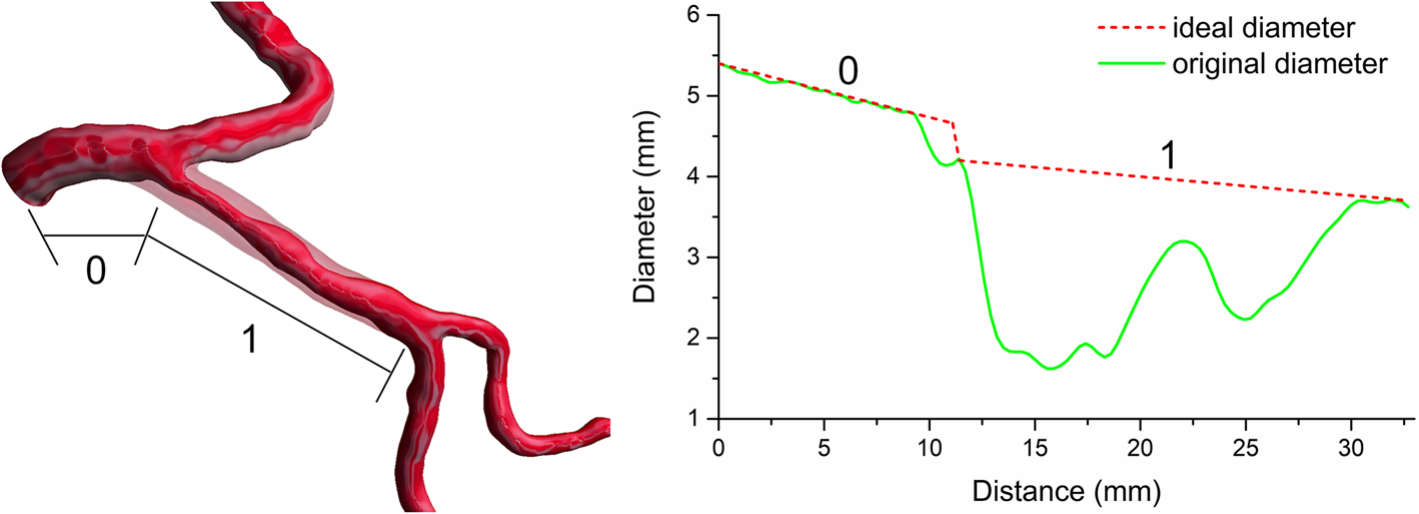
(Left) A partial vessel with a long lesion (solid) and ideal lumen (transparent), which are involved with the father vessel (0) and one of the daughter vessels (1). (Right) The corresponding diseased diameter (green) and ideal diameter (dashed red)

Based on the original diameter and the ideal diameter, the stenosis rate $S_{j}$ can be computed. The original diameter will be replaced using a repaired diameter during flow distribution when stenosis ($S_{j} \ge \beta$, a threshold $\beta$ can be set to 5%) is detected in the first point of daughter vessels. Figure 8 displays a hybrid model superimposed by the ideal model, the patient model and the diameter profile of corresponding models.

### 3D CFD model

Using the open‐source library snappyHexMesh, a 3D mesh representing the coronary artery tree is generated with millions of vertices and elements as the computing model. For 353 left coronary arteries, the average number of mesh elements is 1.46 ± 0.37 million. The average number is 0.89 ± 0.23 million for 69 right coronary arteries. Patient-specific boundary conditions are coupled on the boundary of the 3D model as well. The vessel wall is assumed to be rigid with a no-slip boundary condition. Blood in the coronary artery is considered an incompressible Newtonian fluid, and flow is assumed laminar. Blood flow is numerically simulated by solving the Navier–Stokes equations in a steady state using the open-source library OpenFOAM v18.04. Navier–Stokes equations are discretized by the finite volume method (FVM) and numerically solved by the ‘SIMPLE’ method in an iterative manner. The second-order implicit method is used to discretize the formulation. Among the 3D CFD results, a small part (5% of total) is used to correct pressure loss, and a large proportion (95% of total) is applied for assessing the performance of the FAST algorithm.

### Overall modeling and solving

**Fig. 9:**
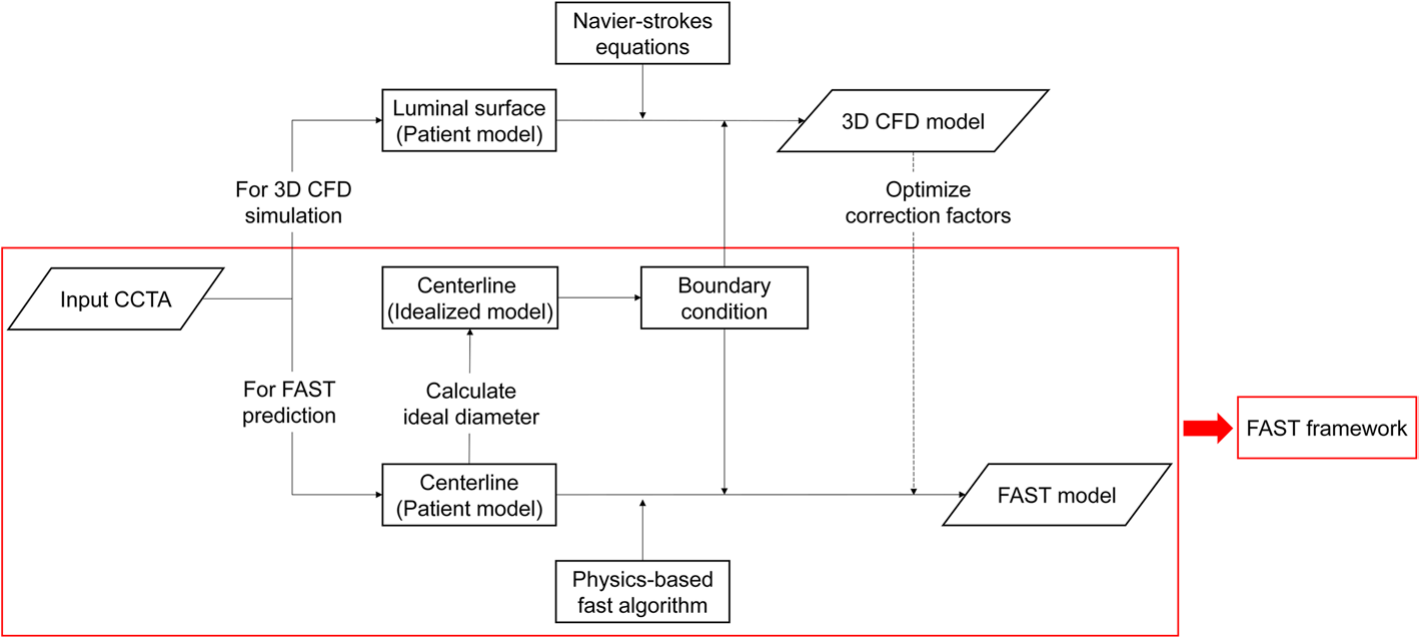
A flowchart representing the steps involved in building a FAST framework. First, a luminal surface model and the centerline of the coronary artery are extracted from the CCTA for the 3D CFD simulation and FAST prediction of blood flow, respectively. Then, an ideal model is constructed to model the appropriate boundary condition. After the 3D CFD calculation is finished, a small part of the result is used for the optimization of correction factors. Next, the FAST method is established by applying the physics-based fast model to the centerline of the coronary artery, combining the boundary condition and corrected factors. Finally, the FAST framework (red frame) can be used to predict hemodynamics by inputting CCTA

**Fig. 10:**
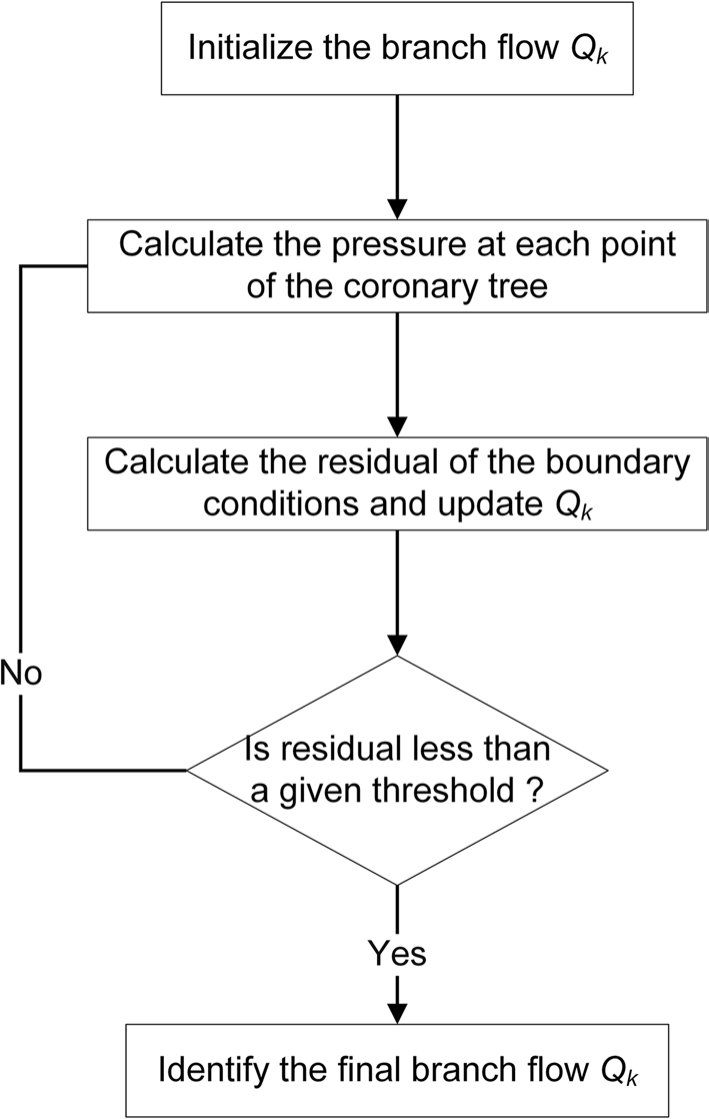
An optimization algorithm flow diagram showing the steps to solve the flow of each branch

The overall modeling and solving process is illustrated in Fig. 9. 3D CFD blood flow simulations and FAST predictions share a pure microvascular resistance boundary condition used for calculations in this work. The core of our FAST framework includes centerline extraction of the coronary artery, boundary condition modeling, and application of the physical-based fast model to the centerline. The last step before the establishment of the FAST algorithm is to compute the correction factors $\left\{ {C_{1} ,C_{2} ,C_{3} ,C_{4} ,C_{5} ,C_{6} } \right\}$. These factors are modeled by solving an optimization problem according to 3D CFD simulation results, which implies that the CFD-based hemodynamic data are used to decide the overdetermined physical constraints to close the modeling by optimizing the parameters $\left\{ {C_{1} ,C_{2} ,C_{3} ,C_{4} ,C_{5} ,C_{6} } \right\}$. After extracting the parameters of $l_{i}$, $d_{i}$, $U_{i}$, $U_{{i,{\text{outlet}}}}$, $U_{{i,{\text{inlet}}}}$, $\lambda_{i}$, $\varsigma_{i}$ and $P_{i}$ at each point of the centerline from CFD-based results, a Trust-region-reflective algorithm [64] is used to solve the unknown variables $\left\{ {C_{1} ,C_{2} ,C_{3} ,C_{4} ,C_{5} ,C_{6} } \right\}$. A group of CFD results is randomly selected from them to determine correction factors of pressure loss in the FAST algorithm. The correction factors do not change during iterative computation after being determined. The Levenberg–Marquardt algorithm [65] is used to address the flow of each branch by satisfying the boundary conditions during calculation, as shown in Fig. 10. The detailed process is as follows:

- Use a constant value (1e−6 m^3^/s) to initialize the branch flow $Q_{k}$;
- Use Eqs. (5) to (12) to calculate the pressure at each point of the coronary tree;
- Use Eq. (14) to calculate the residual of the boundary conditions;
- Use the Levenberg–Marquardt algorithm to update the branch flow $Q_{k}$ and minimize the residual;
- Repeat steps 2–4 until the residual is less than the given threshold.

After the calculation is converged, the blood flow and pressure can be acquired at each point of the coronary centerline. Subsequently, the other quantities can be derived according to the solved blood flow and pressure as well, such as FFR.

## Data Availability

The data presented in this study are available on request from the corresponding author.

## Abbreviations

CABG: Coronary artery bypass graft
CAD: Coronary artery disease
CCTA: Coronary computed tomography angiography
CFD: Computational fluid dynamics
CI: Confidence interval
CT-FFR: Computed tomography fractional flow reserve
CVD: Cardiovascular disease
D: Diagonal
FAST: Fast novel physics-based model
FFR: Fractional flow reserve
FFR_CFD_: 3D CFD-based approach FFR
FFR_FAST_: physics-based fast FFR
FVM: Finite volume method
ICA: Invasive coronary angiogram
LAD: Left anterior descending
LCx: Left circumflex
LVMM: Left ventricular myocardial mass
ML: Machine learning
NPV: Negative predictive value
NS: Navier–Stokes
OM: Obtuse marginal
PCI: Percutaneous coronary intervention
PDA: Posterior descending artery
PPV: Positive predictive value
RCA: Right coronary artery
